# Study protocol: multicenter double-blind, randomized, placebo-controlled trial of rituximab for the treatment of childhood-onset early-stage uncomplicated frequently relapsing or steroid-dependent nephrotic syndrome (JSKDC10 trial)

**DOI:** 10.1186/s12882-019-1470-3

**Published:** 2019-08-02

**Authors:** China Nagano, Mayumi Sako, Koichi Kamei, Kenji Ishikura, Hidefumi Nakamura, Koichi Nakanishi, Takashi Omori, Kandai Nozu, Kazumoto Iijima

**Affiliations:** 1 Department of Pediatrics, Kobe University Graduate School of Medicine, 7-5-1 Kusunoki-cho, Chuo-ku, Kobe, Hyogo 650-0017 Japan; 2 Division for Clinical Trials, Department of Clinical Research Promotion, Clinical Research Center, National Center for Child Health and Development, Tokyo, Japan; 3 Division of Nephrology and Rheumatology, National Center for Child Health and Development, Tokyo, Japan; 4 Clinical Research Center, National Center for Child Health and Development, Tokyo, Japan; 5 Department of Child Health and Welfare (Pediatrics), Graduate School of Medicine, University of the Ryukyus, Okinawa, Japan; 6 Clinical and Translational Research Center, Kobe University Hospital, Kobe, Japan

**Keywords:** Frequently relapsing nephrotic syndrome, Steroid-sensitive nephrotic syndrome, Rituximab

## Abstract

**Background:**

Eighty percent of children with idiopathic nephrotic syndrome respond well to steroid therapy, but up to 50% of patients with steroid-sensitive nephrotic syndrome exhibit frequently relapsing (FRNS) or steroid-dependent nephrotic syndrome (SDNS). Several studies identified the chimeric anti-CD20 monoclonal antibody rituximab as an effective treatment for patients with complicated FRNS/SDNS. Recent studies suggested that rituximab could also be a first-line treatment for early-stage uncomplicated FRNS/SDNS, although further studies are required to confirm its efficacy and safety.

**Methods/design:**

We are conducting a multicenter, double-blind, randomized placebo controlled trial to investigate the efficacy and safety of rituximab for the treatment of childhood-onset early-stage uncomplicated FRNS/SDNS. Patients will be allocated to receive two 375 mg/m^2^ doses (maximum dose: 500 mg) of either rituximab or placebo. Investigators are permitted to request the disclosure of a subject’s allocation code if he or she exhibits treatment failure. Additionally, if placebo-treated subjects display early relapse (a sign of treatment failure), they have the option to receive rituximab in an unblinded phase. The primary endpoint is relapse-free survival during the observation period.

**Discussion:**

The results will provide important data on the use of rituximab for patients with uncomplicated FRNS/SDNS. In the future, rituximab treatment will enable most patients with uncomplicated FRNS/SDNS to discontinue or reduce steroid therapy without relapse, and it is possible that rituximab could represent an immunosuppressive therapy for these diseases.

**Trial registration:**

This trial was prospectively registered to the JMACCT Clinical Trials Registry on September 6, 2018 (Trial ID: JMA-IIA00380).

## Background

Nephrotic syndrome (NS) describes a clinical presentation of edema, proteinuria, hypoalbuminemia, and hyperlipidemia. NS affects 1 to 3 per 100,000 children less than 16 years of age [1]. In Japan, the estimated incidence of INS is 6.49 cases per 100,000 children annually [2]. Multiple pathogenic mechanisms that ultimately disrupt the glomerular filtration barrier have been identified.

The majority of children with idiopathic NS (80–90%) are steroid-sensitive, with their proteinuria normalizing within 4 weeks of daily oral corticosteroid administration [3]. Most steroid-sensitive patients (90%) have minimal-change NS, whereas most steroid-resistant patients (80.5–94.4%) have focal segmental glomerulosclerosis or mesangioproliferative glomerulonephritis. Approximately 30% of patients experience only one attack before a subsequent cure after the first course of therapy, 10–20% of patients have three or four steroid-responsive episodes before a permanent cure, and the remaining 30–50% of patients are frequent relapsers making them steroid-dependent.

The long-term prognosis for most children with steroid-sensitive nephrotic syndrome is complete resolution of their disease over time and maintenance of normal kidney function; therefore, limiting the long-term adverse effects of treatment is an important objective. Children with frequently relapsing NS (FRNS) or steroid-dependent NS (SDNS) require prolonged corticosteroid therapy, which is associated with significant adverse effects, including impaired linear growth, behavioral changes, obesity, Cushing’s syndrome, hypertension, ophthalmological disorders, impaired glucose tolerance, and reduced bone mineral density. Adverse effects may persist into adulthood in young people, who continue to relapse after puberty [4].

To reduce the risks of corticosteroid-related adverse effects, children with FRNS or SDNS (FRNS/SDNS) may require other agents. The Japanese Society for Pediatric Nephrology treatment guidelines for idiopathic NS in children recommend cyclosporine (3–6 mg/standard body weight [kg]/day for 2 years), cyclophosphamide (2–2.5 mg/standard body weight [kg]/day for 8–12 weeks), and mizoribine (high dose, 7–10 mg/standard body weight [kg]/day) as immunosuppressive drugs for FRNS. Given the toxicity of these agents, alternative treatment options must be investigated. Rituximab, a chimeric monoclonal anti-CD20 antibody, is increasingly being used as a steroid-sparing treatment option for children with idiopathic NS. The 2012 Kidney Disease: Improving Global Outcomes clinical practice guidelines on glomerulonephritis introduced rituximab as a treatment option for childhood-onset complicated FRNS/SDNS, and its efficacy and safety for these conditions have been established [5–7].

Rituximab was approved in Japan by the Ministry of Health, Labor, and Welfare for complicated FRNS/SDNS on August 29, 2014 based on the results of RCRNS-01 [5] and RCRNS-02. In these studies, some patients who can discontinue immunosuppressive agents before rituximab treatment may achieve long-term remission after rituximab treatment without any treatment. It is possible that rituximab could weaken disease activity and alter long-term prognosis in these patients [8]. It is likely that rituximab can be used as a first-line drug to treat cases of uncomplicated SDNS. Indeed, rituximab has been used as a first-line treatment for uncomplicated SDNS at many centers in European countries [9].

Only a few trials have assessed the use of rituximab in early-stage uncomplicated SDNS. Recently, an open-label, noninferiority, randomized controlled trial tested whether rituximab is noninferior to steroids in maintaining remission in childhood uncomplicated SDNS. The results from this trial indicated that rituximab allows the complete withdrawal of steroids in patients with childhood SDNS without adversely affecting clinical outcomes and that the drug has an acceptable short-term adverse event profile [10]. One clinical trial indicated that rituximab is more effective than tacrolimus over a 12-month period in maintaining disease remission and minimizing corticosteroid exposure [11]. Despite these results, additional data are needed to inform clinical practice. We believe that rituximab is likely to be a standard treatment for frequent relapse or steroid dependence, which prompted us to create this protocol. Currently, the use of rituximab in Japan for uncomplicated FRNS/SDNS patients requires approval from the Ministry of Health, Labor and Welfare. The proposed clinical trial seeks to establish evidence for the use of rituximab for first-line treatment of uncomplicated FRNS/SDNS patients.

## Methods/design

### Aims

In this trial, we aim to evaluate the efficacy and safety of two 375 mg/m^2^ doses of IDEC-C2B8 (rituximab: a monoclonal antibody directed against the CD20 differentiation antigen expressed on the surface of B-cells) given at weekly intervals in patients with childhood-onset early-stage uncomplicated FRNS/SRNS.

We also aim to verify the duration of relapse-free survival during the unblinded observation period, clarify the IDEC-C2B8 blood concentration and human anti-chimeric antibody (HACA) production and peripheral blood B cell numbers, and assess the relationship between recurrence and adverse events.

### Study design (Fig. 1)

We are conducting a multicenter, double-blind, randomized, placebo-controlled trial of IDEC-C2B8 for the treatment of childhood-onset early-stage uncomplicated FRNS/SRNS. In total, 40 patients (20 in each group) from 12 institutions in Japan will be enrolled in this study.

**Fig. 1:**
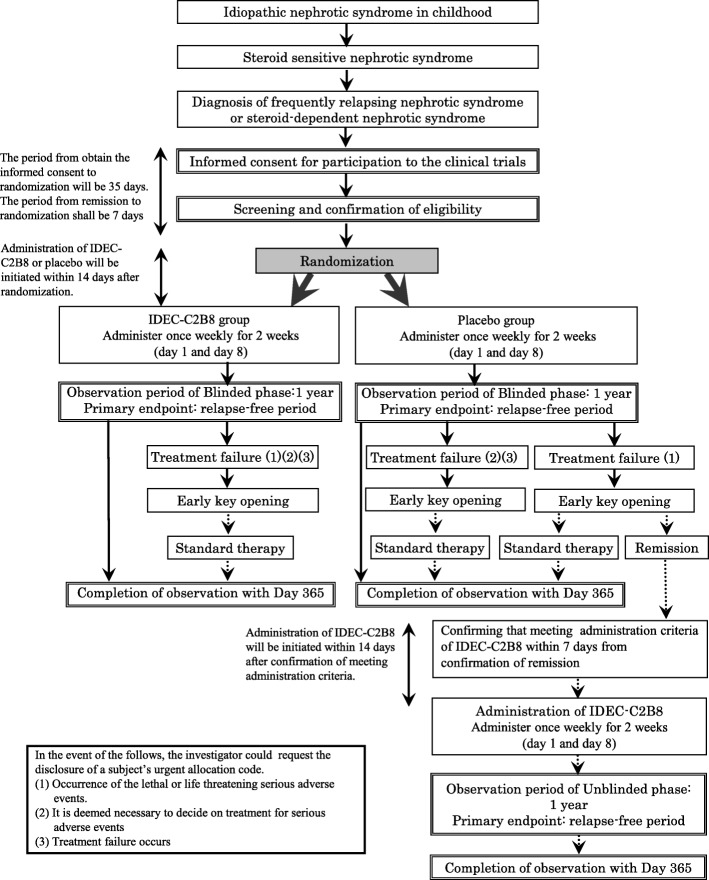
Flow diagram of the clinical trial set-up. The trial is a multicenter, double-blind, randomized, placebo-controlled study. After obtaining informed consent, randomization will be conducted. If subjects in the placebo group wish to receive IDEC-C2B8 after the initial phase, they will receive the drug in an unblinded phase

However, if subjects in the placebo group develop treatment failure [1] and wish to receive IDEC-C2B8 after the randomized blinded phase, they will be eligible for treatment with the drug during the unblinded phase.

#### Clinical trial diagram (Figs. 2 and 3)

The clinical trial period for each patient will begin on the date consent is obtained and end on the date of completion of the observation period. However, subjects who desire to transition to the unblinded phase and receive IDEC-C2B8 after the initial blinded phase will remain in the study until the end date of the unblinded observation period. The day on which the first dose of the investigational drug is administered during the blinded period will be set as “Blinded Day1.” The day on which the first dose of investigational drug is administered during the unblinded period will be set as “Unblinded Day 1.”

**Fig. 2:**
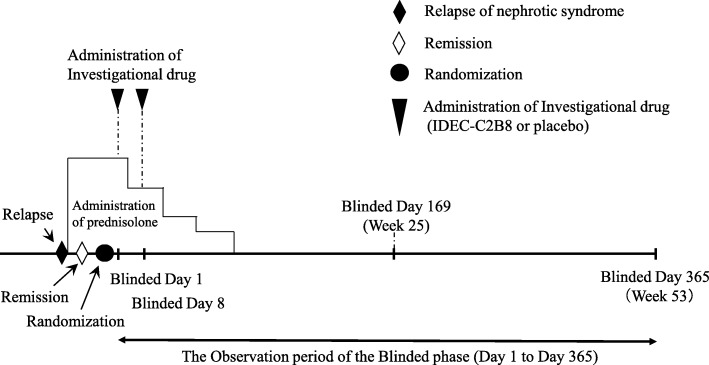
Dosage regimen (blinded period). Investigators will administer the first dose of the investigational drug within 14 days after the date of randomization (the date the first dose of the investigational drug is administered is set as Blinded Day 1). The investigational drug will be administered via two 375-mg/m^2^ doses (maximum dose: 500 mg) separated by 1 week (Blinded Days 1 and 8)

**Fig. 3:**
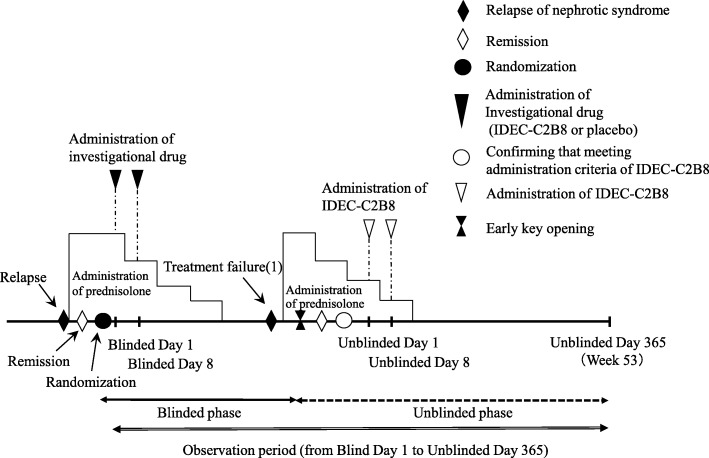
Dosage regimen (unblinded period). Investigators will administer the first dose of IDEC-C2B8 within 14 days after the date of allocation (the date the first dose of IDEC-C2B8 is administered is set as Unblinded Day 1). IDEC-C2B8 will be administered via two 375-mg/m^2^ doses (maximum dose: 500 mg) separated by 1 week (Unblinded Days 1 and 8)

#### Definition of treatment failure

In this study, treatment failure is defined as follows:

- Treatment failure (1): Relapse occurring prior to Week 25 (Blinded Day 168).
- Treatment failure (2): Diagnosis of frequent relapse (two or more relapses)/steroid dependence between Weeks 25 (Blinded Day 169) and 53 (Blinded Day 365).
- Treatment failure (3): Diagnosis of steroid resistance after the blinded observation period.

### Study patients

The inclusion criteria for patients are as follows:

1. Fulfill the diagnostic criteria of idiopathic NS (the International Study of Kidney Disease in Children [ISKDC] idiopathic NS diagnostic criteria are used as a reference during the patient’s initial diagnosis).
2. Younger than 18 years at the time of onset of idiopathic NS.
3. Diagnosis of frequent relapse (two or more relapses within 6 months after initial remission or four or more relapses within any 12-month period) or steroid dependence (two consecutive relapses during steroid therapy dose reduction or within 2 weeks of discontinuation of steroid therapy), as well as confirmation of those dates of NS relapse prior to assignment.
4. No history of treatment with any immunosuppressant for NS.
5. Observation of steroid sensitivity during the treatment of relapse immediately prior to assignment.
6. CD20-positive cell count of at least 5/μL in peripheral blood.
7. Ability to stay in the hospital overnight on all days of planned investigational drug administration.
8. Provision of consent by the patient (aged 20 years or older) or a legal representative (parents or legal guardians) using a document approved by the institutional review board after receiving an adequate explanation regarding the implementation of this clinical trial.

The exclusion criteria are as follows:

1. Diagnosis of SRNS.
2. Diagnosis of nephritic or secondary NS.
3. History of severe infections such as pulmonary tuberculosis, deep mycosis, human immunodeficiency virus (HIV), hepatitis C virus (HCV), or hepatitis B virus (HBV) or other active viral infections.
4. History of angina pectoris, cardiac failure, myocardial infarction, or serious arrhythmia.
5. Receipt of a live vaccine within 4 weeks prior to assignment.
6. Poorly controlled hypertension.
7. Impaired renal function.
8. Diagnosis of autoimmune diseases, IgA vasculitis, or systemic lupus nephritis.
9. History of malignant tumors.
10. History of organ transplantation (excluding corneal and hair transplants).
11. Allergy to methylprednisolone, acetaminophen, or d-chlorpheniramine maleate.
12. Any of the following abnormal clinical laboratory values at the time of registration: leukocytes, < 3000/μL; neutrophils, < 1500/μL; platelets, < 50,000/μL; severe liver dysfunction (if younger than 21 years: glutamic-oxaloacetic transaminase [GOT] or glutamic pyruvic transaminase [GPT] levels equal to or greater than 2.5× the upper limit of normal determined by the “Reference Intervals of Clinical Tests in Children Determined by a Latent Reference Value Extraction;” 21 years or older: GOT or GPT levels equal to or greater than 2.5× the upper limit of normal of each medical facility); hepatitis B surface (HBs) antigen, HBs antibody, hepatitis B core antibody, or HCV antibody positivity; HIV antibody positivity.
13. Prior receipt of monoclonal antibodies of any type (mouse, rat, chimeric or human).
14. Prior receipt of other investigational drugs within 6 month before assignment or participation in other studies at the time of assignment.
15. Pregnancy, breastfeeding, or ability to become pregnant and refusal to use contraception during the study (confirmation by serum human chorionic gonadotropin test is required during screening).
16. Assessed to be unfit for participation by the investigators.

### Study medication

The investigational drugs used in the study are a test drug and a control drug. The two drugs are impossible to differentiate from each other based on their external appearances.

The open-label study drug (IDEC-C2B8) after the initial phase is identical to the study drug used in the blinded phase excluding the packaging and labeling.

Zenyaku Kogyo Co., Ltd. has provided the study drugs of both test drug and placebo, and subsequently distributed to institutes that have enrolled patients in this trial.

#### Overview of the test drug

1. Development code name and International non-proprietary names
  Development code name: IDEC-C2B8.
  International non-proprietary names: rituximab.
  [Japanese accepted names] (English name) rituximab (genetic recombination)
2. Description: Chimeric anti-CD20 monoclonal antibody with mouse anti-CD20 antibody variable regions and human constant regions (IgG1κ) (molecular weight: 144,510 Da)
3. Special notes: IDEC-C2B8 has been approved for the treatment of CD20-positive B-cell lymphoproliferative disease, CD20-positive B cell non-Hodgkin’s lymphoma, ANCA-related vasculitis, chronic idiopathic thrombocytopenic purpura, Wegener’s granulomatosis, systemic lupus erythematodes, and complicated steroid dependent / frequently relapsing nephrotic syndrome in Japan and it is already on the market. The commercial products are designated as biological products.

### Prohibited combination therapy or concomitant medication

Combination therapy using following drugs is prohibited during the period of clinical trial (the consent date up to the end of the observation period).

1. Commercial rituximab.
2. Immunosuppressants or alkylating agents having an immunosuppressive effect, unless cases determined to be treatment failure.
3. Plasma exchange.
4. Live vaccines.
5. Other investigational drugs or unapproved drugs in Japan.

### Screening

When investigators observe a recurrence of NS in study candidate patients, they describe this clinical trial to the relevant subjects and obtain their written consent to participate in the trial. After consent is obtained, screening tests are performed to verify eligibility as a subject. If the eligibility of patients for this trial is confirmed after the screening period, the patients will be assigned within 7 days of confirmation of remission after relapse.

### Randomization

Patients will be randomly assigned to the IDEC-C2B8 or placebo group at a 1:1 ratio. Randomization will be performed using the stratification permuted block method. The assignment adjustment factors are frequent recurrence/steroid dependency. All participating physicians and other investigators will remain blinded to the trial results until follow-ups are completed.

### Binding

JSKDC10 is a placebo-controlled double-blind clinical trial. Registration and assignment are done using the data center assignment program. Only drug numbers assigned to the patient will be displayed to the data center personnel.

The allocation code for the allocation program is entered by the registration and allocation personnel. However, blinding is protected as the assignment code corresponding to the drug number and will not be accessed except at the entry point.

### Procedures

The investigators have to do the first administration of the investigational drug within 14 days after assignment (the first administration date of the investigational drug is set to be Blinded Day 1, week 1). The investigational drug or placebo will be administered using two 375 mg/m^2^ doses (maximum dose: 500 mg) at weekly intervals (Blinded Day 1 and 8). Premedication consisting of acetaminophen, d-chlorpheniramine maleate and methylprednisolone are to be given 30 min prior to the investigational drug infusions to decrease the incidence and severity of infusion reactions. Prednisolone treatment for relapse during the observation period will follow the ISKDC regimen.

### Blood sampling (Tables 1 and 2)

During the clinical trial period, investigators will perform observation, examination, and survey according to a predetermined schedule in this protocol. On all days of investigational drug administration, blood samples are taken immediately prior to administration.

**Table 1:**
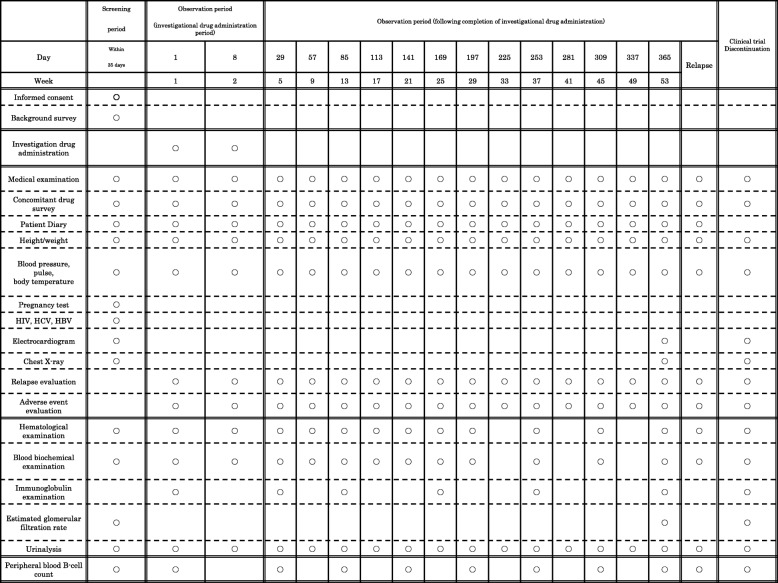
Schedule (Blinded period)

**Table 2:**
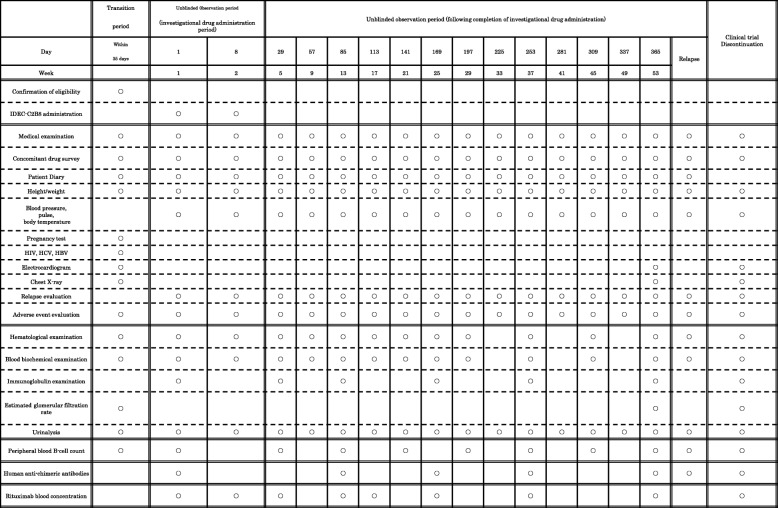
Schedule (Unblinded period)

### Conditions for urgent allocation code disclosure

The investigator may request disclosure of the subject’s emergency allocation code if any of the following situations apply:

1. The subject experiences a severe adverse event that leads to death or life-threatening.
2. The subject experiences a serious adverse event, and the information is judged to be essential for deciding on the treatment of other subjects.
3. Treatment failure occurs.

### Outcomes

The primary endpoint is the time of relapse-free survival during the blinded observation period (days 1–365), assessed as the period from the date of assignment/allocation until that of the first relapse following the commencement of investigational drug administration. Secondary endpoints are the time to treatment failure, total steroid dosage, and changes in the number of B-cells in peripheral blood. Other endpoints are the time of relapse-free survival during the unblinded observation period (days 1–365), IDEC-C2B8 blood concentration, HACA production ratio, and adverse events. Adverse events will be recorded throughout the trial period and assessed using the Japan Clinical Oncology Group (JCOG) Japanese-language version (September 12, 2017) of the Common Terminology Criteria for Adverse Events version 4.0.

### Sample size

A Kaplan–Meier plot of the open-label randomized controlled trial reported from the previous study [6] revealed relapse-free rates at 1 year of 0.66 in the rituximab group and 0 in the steroid alone group. Based on these results, assuming an exponential distribution for relapse-free survival and assuming that the relapse-free rate in 1 year of steroid treatment alone was 0.01 as the placebo group, the hazard rates were 0.42 for the rituximab group and 4.60 for the steroid monotherapy group, resulting in a hazard ratio of 0.09. If the registration period is 17 months, and the observation period of the blinded phase is 12 months, resulting in a total clinical trial period of 29 months, to test the superiority using a significance level of 5% (two-sided) and a detection power of 80%, the required number of the patients was estimated to be 10 per group. At 1-year relapse-free rates of 0.50, 0.40, and 0.30 in the rituximab group, the required numbers of the patients calculated under similar conditions were 12, 13, and 16 per group, respectively. Considering the possibility of withdrawal of consent after participation in the study or the loss of subjects to follow-up and uncertainty for the estimation of 1-year relapse-free rates in placebo group, we set the target sample number to 40 patients (20 in each group).

### Statistical analysis

The objective of the primary analysis (the primary endpoint) in this clinical trial is to examine whether the IDEC-C2B8 group has significantly longer relapse-free survivals than the placebo group. The “relapse-free survival between the two groups will be equal” null hypothesis will be tested using the stratified log-rank test with the subject population receiving at least one dose of the investigational drug administration and measured the data for the assessment of primary endpoint (full analysis set). If the relapse-free survival is shorter in the IDEC-C2B8 group than in the placebo group, the test is one-sided because statistical significance is not important. One-sided significance level was set at 2.5%. If recurrence free survival is significantly longer in the IDEC-C2B8 group than in the placebo group, it can be concluded that IDEC-C2B8 is a useful treatment. Cumulative relapse curves and estimation of time to relapse will be performed using the Kaplan-Meier method and stratified log rank test of time to treatment failure.

The following analyses for the secondary and other endpoints are planned.

The total steroid dose in the blinded observation period will be analyzed using the analysis of covariance.

Median of the time to normalization of peripheral blood B-cells and its 95% confidence interval will be estimated using the Kaplan-Meier method.

The relationships of normalization of the number of peripheral blood B-cells with the presence or absence of relapse and with adverse events will be evaluated in each blinded observation period using Fisher’s exact test.

The proportion for the HACA production in each period with its 95% confidence interval will be estimated and the relationship of the presence or absence of HACA with adverse events will be evaluated in each period by Fisher’s exact test. IDEC-C2B8 blood concentration will be summarized to maximum blood concentration (C_max_), half-life period (T_1/2_), mean residence time (MRT) and area under the concentration-time curve (AUC), and the mean and standard deviation of these parameters will be calculated.

### Participating hospitals

Dokkyo Medical University Hospital, Japan.

Nihon University Itabashi Hospital, Japan.

National Center for Child Health and Development, Japan.

Tokyo Metropolitan Children’s Medical Center, Japan.

Yokohama City University Medical Center, Japan.

Shiga University of Medical Science Hospital, Japan.

Osaka University Hospital, Japan.

Kobe University Graduate School of Medicine, Japan.

Hyogo Prefectural Kobe Children’s Hospital, Japan.

Wakayama Medical University, Japan.

Kurume University Hospital, Japan.

Saga University Hospital, Japan.

### Coordinating center

The coordinating center will provide various clinical trial coordination activities, including protocol preparation support and project management, to facilitate the activities of the clinical trial coordination committee.

## Discussion

Herein, the protocol for a multicenter, double-blind, randomized placebo controlled trial to investigate the efficacy and safety of rituximab for the treatment of childhood-onset early-stage uncomplicated FRNS/SDNS is described. This trial has several strengths.

First, it is a multicenter and double-blind protocol. To date, only two major reports have assessed the use of rituximab in early-stage uncomplicated SDNS. Ravani et al. conducted a multicenter, open-label, randomized, control trial [10] and Basu et al. conducted a single-center, parallel-arm, open-label, randomized, clinical trial [11]. Compared with these previous reports, our trial is of high quality as clinical research. As the efficacy and safety of drugs can vary depending on geographic location, to evaluate the efficacy of rituximab, a multicenter trial of a Japanese population is therefore needed.

Second, this study also features an unblinded phase. In this manner, it will be possible to clarify the effects and side effects of rituximab by comparing the efficacy and safety during the placebo treatment phase and the active drug treatment phase. Additionally, this study will increase the treatment options for children who experience treatment failure.

Third, the protocol alleviates concerns about treatment compliance because the medication is administered via intravenous infusion. As current recommended immunosuppressive drugs have serious side effects, and it is necessary to continue oral administration each day or every other day, a lack of treatment compliance is common.

Fourth, several clinical benefits may be observed in the rituximab group at the end of the study period. One potential benefit is good renal function because of the absence of calcineurin inhibitor-associated renal vasoconstriction and chronic nephrotoxicity. Other possible benefits include catch-up growth and weight loss because of corticosteroid sparing.

Finally, this trial has multiple secondary endpoints. Assessing the relationship between recurrence and the number of B cells may help to determine the pathology of NS. However, our study has limitations, including a small sample size and its short-term duration.

In conclusion, we are conducting a multicenter, double-blind, randomized placebo controlled trial to evaluate the efficacy and safety of rituximab for early-stage uncomplicated FRNS/SDNS. The results of this study may support the use of rituximab as a first-line corticosteroid-sparing therapy for patients with early-stage uncomplicated FRNS/SDNS.

## Data Availability

Not applicable for this publication.

## Abbreviations

AUC: Area under the concentration-time curve
C_max_: maximum blood concentration
FRNS: Frequently relapsing nephrotic syndrome
GOT: Glutamic-oxaloacetic transaminase
GPT: Glutamic pyruvic transaminase
HACA: Human anti-chimeric antibody
HBs: Hepatitis B surface
HCV: Hepatitis C virus
HIV: Human immunodeficiency virus
IgA: Immunoglobulin A
IgG: Immunoglobulin G
ISKDC: International Study of Kidney Disease in Children
JCOG: the Japan Clinical Oncology Group
MRT: Mean residence time
NS: Nephrotic syndrome
SDNS: Steroid-dependent nephrotic syndrome
SRNS: Steroid-resistant nephrotic syndrome
T_1/2_: half-life period

## References

[1] Mc Kinney PA, Feltbower RG, Brocklebank JT, Fitzpatrick MM (2001). Time trends and ethnic patterns of childhood nephrotic syndrome in Yorkshire, UK. Pediatric nephrology.

[2] Kikunaga Kaori, Ishikura Kenji, Terano Chikako, Sato Mai, Komaki Fumiyo, Hamasaki Yuko, Sasaki Satoshi, Iijima Kazumoto, Yoshikawa Norishige, Nakanishi Koichi, Nakazato Hitoshi, Matsuyama Takeshi, Ando Takashi, Ito Shuichi, Honda Masataka (2016). High incidence of idiopathic nephrotic syndrome in East Asian children: a nationwide survey in Japan (JP-SHINE study). Clinical and Experimental Nephrology.

[3] Hahn D, Hodson EM, Willis NS, Craig JC. Corticosteroid therapy for nephrotic syndrome in children. The Cochrane Database Syst Rev. 2015;(3):CD001533. 10.1002/14651858.CD001533.pub5 PMC7025788 25785660

[4] Kyrieleis HA, Löwik MM, Pronk I, Cruysberg HR, Kremer JA, Oyen WJ (2009). Long-term outcome of biopsy-proven, frequently relapsing minimal-change nephrotic syndrome in children. Clin J Am Soc Nephrol.

[5] Iijima K, Sako M, Nozu K, Mori R, Tuchida N, Kamei K (2014). Rituximab for childhood-onset, complicated, frequently relapsing nephrotic syndrome or steroid-dependent nephrotic syndrome: a multicentre, double-blind, randomised, placebo-controlled trial. Lancet.

[6] Ravani P, Bonanni A, Rossi R, Caridi G, Ghiggeri GMAnti-CD20 Antibodies for Idiopathic Nephrotic Syndrome in Children. Clin J Am Soc Nephrol. 2016;11(4):710–20. 10.2215/CJN.08500815 PMC4822672 26585985

[7] Ravani P, Magnasco A, Edefonti A, Murer L, Rossi R, Ghio L (2011). Short-term effects of rituximab in children with steroid- and calcineurin-dependent nephrotic syndrome: a randomized controlled trial. Clin J Am Soc Nephrol.

[8] Kamei K, Ishikura K, Sako M, Aya K, Tanaka R, Nozu K (2017). Long-term outcome of childhood-onset complicated nephrotic syndrome after a multicenter, double-blind, randomized, placebo-controlled trial of rituximab. Pediatric nephrology.

[9] Vivarelli M, Deschênes G Idiopathic Nephrotic syndrome Working Group Report, September 2016. http://espn-online.org/images/IdiopathicNephroticsyndromeWorkingGroupReport2016.pdf. Accessed 3 Apr 2017.

[10] Ravani P, Rossi R, Bonanni A, Quinn RR, Sica F, Bodria M (2015). Rituximab in Children with Steroid-Dependent Nephrotic Syndrome: A Multicenter, Open-Label, Noninferiority, Randomized Controlled Trial. J Am Soc Nephrol.

[11] Basu B, Sander A, Roy B, Preussler S, Barua S, TKS M (2018). Efficacy of Rituximab vs Tacrolimus in Pediatric Corticosteroid-Dependent Nephrotic Syndrome: A Randomized Clinical Trial. JAMA Pediatr.

